# pH-Responsive Nanogels Generated by Polymerization-Induced Self-Assembly of a Succinate-Functional Monomer

**DOI:** 10.1021/acs.macromol.4c00427

**Published:** 2024-04-08

**Authors:** Ruiling Du, Lee A. Fielding

**Affiliations:** † Department of Materials, School of Natural Sciences, University of Manchester, Oxford Road, Manchester M13 9PL, U.K.; ‡ Henry Royce Institute, The University of Manchester, Oxford Road, Manchester M13 9PL, U.K.

## Abstract

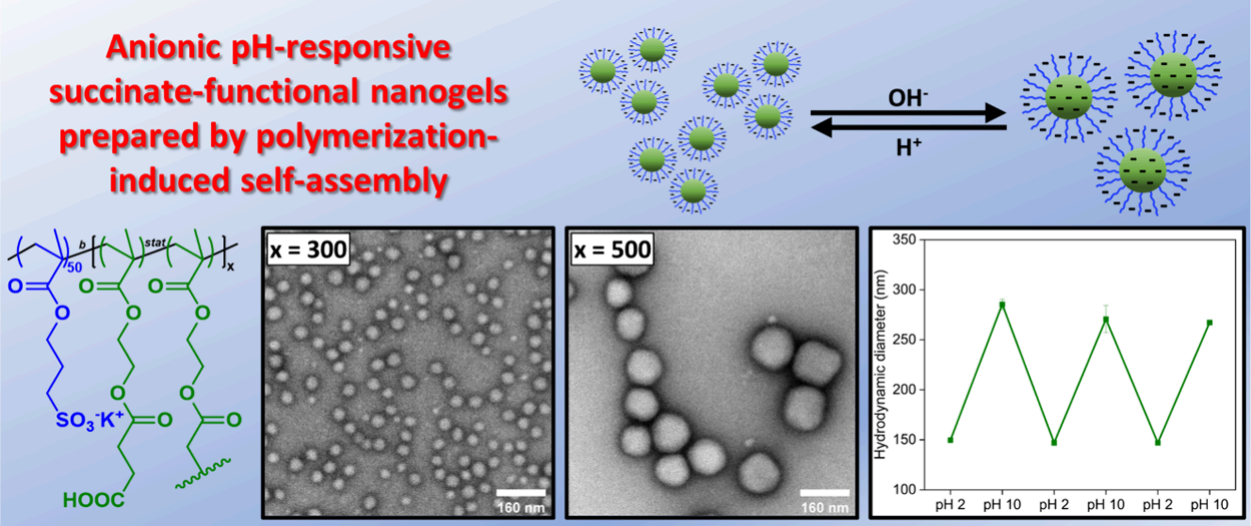

Colloidal nanogels formed from a pH-responsive poly(succinate)-functional core and a poly(sulfonate)-functional corona were prepared via a previously unreported reversible addition–fragmentation chain-transfer (RAFT)-mediated aqueous emulsion polymerization-induced self-assembly (PISA) route. Specifically, a poly(potassium 3-sulfopropyl methacrylate) (PKSPMA_50_) macromolecular chain-transfer agent (macro-CTA) was synthesized via RAFT solution polymerization followed by chain-extension with a hydrophobic, carboxylic acid-functional, 2-(methacryloyloxy) ethyl succinate (MES) monomer at pH 2. Colloidal nanoparticles with tunable diameters between 66 to 150 nm, depending on the core composition, and narrow particle size distributions were obtained at 20% w/w solids. Well-defined pH-responsive nanogels that swell on increasing the pH could be prepared even without the addition of a cross-linking comonomer, and introducing an additional cross-linker to the core led to smaller nanogels with lower swelling ratios. These nanogels could reversibly change in size on cycling the pH between acidic and basic conditions and remain colloidally stable over a wide pH range and at 70 °C.

## Introduction

Nanogels are polymer nanoparticles that can swell and generally range in size from tens to hundreds of nanometers in diameter.^1^ They can be responsive to environmental stimuli such as pH,^2^ solvent quality,^3^ and temperature,^4^ and typically change size in response to these conditions. The properties of nanogels can be fine-tuned across a wide parameter space, including size,^5^ charge,^6^ stimuli-responsive features,^7^ architecture,^8^ and softness.^9^ Therefore, these soft nanomaterials have found extensive applications in the field of biology and medicine, ranging from bioimaging^10^ and photosensitization^11^ to molecular delivery.^12^

Nanogels can be prepared through a variety of methods including cross-linking of functionalized macromolecular precursors^2^ or direct monomer polymerization.^13^ The latter synthetic approach combines both polymerization and nanogel formation into a single, streamlined process, which is typically achieved using free radical polymerization. Controlled free radical polymerizations to form self-assembled block copolymer nanoparticles offers the benefits of traditional free radical polymerization and can yield well-defined cross-linked nanoparticles with controlled nanostructures,^14^ surface chemistries,^15^ and uniform size dispersity^16^ without the need for surfactants or potentially toxic solvents. Reversible addition–fragmentation chain transfer (RAFT)-mediated polymerization-induced self-assembly (PISA) is widely recognized for its high tolerance to a range of functional monomers^17^ and reaction conditions,^18^ making it the most versatile PISA approach for many researchers in the field.^19−21^ In this process, a solvophilic homopolymer is typically chain-extended with monomer(s) in a selective solvent for the second block, resulting in nanoparticle formation during polymerization.

Nanogel synthesis by RAFT-mediated PISA in water is currently dominated in the literature by RAFT aqueous dispersion polymerization,^22^ where a soluble monomer forms an insoluble polymer during polymerization. In contrast, emulsion polymerization requires the use of water-immiscible core-forming monomers and as such preparing swellable nanogels through an emulsion polymerization route typically requires the use of a responsive comonomer.^23^ Similarly, while there have been many examples of PISA-derived nanogels with thermoresponsive behavior,^24−26^ there are relatively limited examples where the core of the nanoparticle provides pH-responsive functionality.^27^ Examples of pH-responsive nanogels in the literature typically are either obtained from modifying existing polymers with groups that are subsequently used to form cross-links,^28^ or based on the insertion of a pH-responsive comonomer into the core block.^23,29^

Nanogels with pH-responsive carboxylic acid functional groups in their cores are of great interest as they are potentially suitable for drug/cargo delivery in the various tissues and cellular compartments in the body.^30^ 2-(Methacryloyloxy)ethyl succinate (MES) is a commercially available vinyl monomer containing a carboxylic group that is potentially of use in bioapplications.^31^ For example, PMES films have been shown to act as protein binders for protein purification by affinity adsorption.^32^ PMES homopolymer has previously been prepared by RAFT solution polymerization^33^ but, to the best of our knowledge, has not been investigated in the context of PISA nor as a nanogel core-forming polymer. Hence, the use of MES as a core-forming monomer in RAFT-mediated aqueous emulsion PISA offers a novel strategy for the formation of pH-responsive nanogels.

Herein, the preparation of amphiphilic diblock copolymer nanogels using MES is reported. Specifically, a poly(potassium 3-sulfopropyl methacrylate) (PKSPMA_50_) macromolecular chain-transfer agent (macro-CTA) was chain-extended with carboxylic acid-functional MES with or without cross-linker (ethylene glycol dimethacrylate, EGDMA), at pH 2 to generate polyacid core, pH-responsive, nanogels. Systematic variation of the degree of polymerization of the PMES core-forming block and degree of cross-linking enabled the synthesis of nanogels with tunable sizes and degree of swelling. Transmission electron microscopy (TEM) and dynamic light scattering (DLS) were used to characterize the particle size and morphology of the resulting nanoparticles, and DLS was further used to monitor the pH- and temperature-responsiveness of the nanogels.

## Results and Discussion

**Figure 1:**
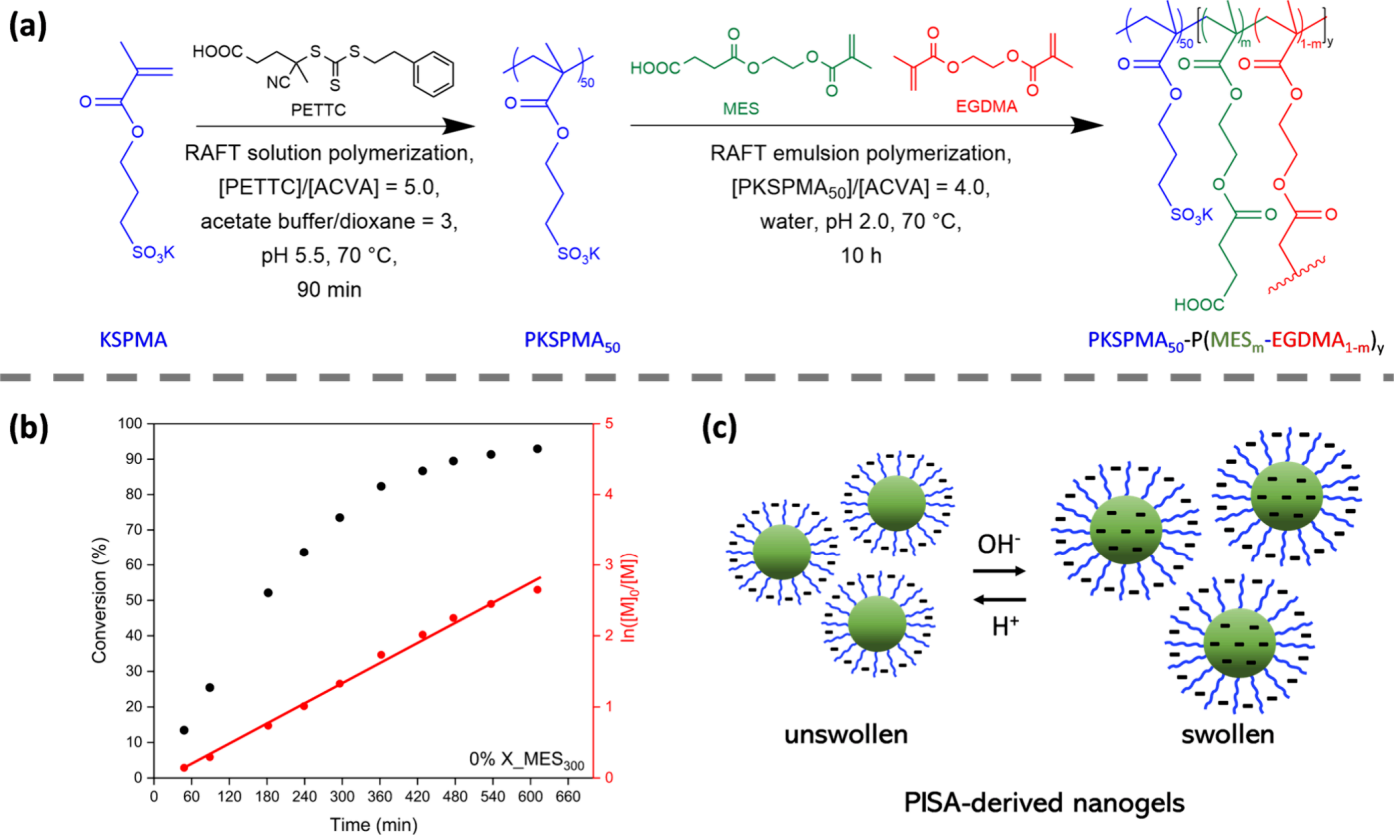
(a) Synthesis of poly(potassium 3-sulfopropyl methacrylate)_50_ (PKSPMA_50_) via RAFT solution polymerization at 70 °C (15% w/w, pH 5.5), followed by RAFT-mediated aqueous emulsion PISA of mono-2-(methacryloyloxy)ethyl succinate (MES) at 70 °C (20% w/w, pH 2.0). (b) Kinetic study for the RAFT emulsion polymerization of MES (target DP 300) using PKSPMA_50_ as a macro-CTA in water at 70 °C (20% w/w, pH 2.0). (c) Schematic representation of pH-responsive nanogel behavior.

Brief details of the syntheses are described below (Figure 1a, see ESI for further details). First, RAFT solution polymerization of KSPMA was conducted in an acetate buffer/dioxane cosolvent at 70 °C using 4-cyano-4-(2-phenylethane sulfanylthiocarbonyl) sulfanylpentanoic acid (PETTC) as a chain-transfer agent (CTA). This afforded a low polydispersity PKSPMA macro-CTA with a mean degree of polymerization (DP) of 50 (Figure S1b). The KSPMA polymerization reaction was quenched at 89% monomer conversion (Figure S1a), to avoid monomer-starved conditions and hence ensure a high degree of RAFT end-group functionalization^34^ PKSPMA_50_ macro-CTA had a molar mass dispersity (*M*_w_/*M*_n_) of 1.13 (Figure S2), which is consistent with previous studies reporting well-controlled RAFT syntheses.^35^ Subsequently, RAFT emulsion polymerization of MES was conducted in water using PKSPMA_50_ as the macro-CTA. As shown in Figure S3, the solubility of MES varies as a function of pH. Hence, polymerizations were conducted at low pH to ensure that MES remained protonated, and the reaction was conducted under emulsion polymerization conditions.

A kinetic study was performed for a target copolymer composition of PKSPMA_50_-PMES_300_ whereby samples were periodically taken from the reaction mixture and analyzed by ^1^H NMR spectroscopy in 80/20% w/w methanol-d_4_/D_2_O (Figure S4). Monomer conversion was measured by integrating the proton signals corresponding to the reactive double bond on MES (5.60–6.25 ppm) and the methylene protons in the α- and β-positions of the carboxyl group on (P)MES (2.50–2.93 ppm). A linear relationship between ln([*M*]_0_/[*M*]) and reaction time confirmed that polymerization of MES was first-order with respect to monomer concentration (Figure 1b), as expected for RAFT polymerizations.^35^

Given that MES contains a potentially hydrolyzable ester linkage, the degree of hydrolysis during polymerization was scrutinized. A new proton signal appeared at 3.65 ppm during the polymerization, indicating that some hydrolysis was indeed occurring (Figure S4). However, the amount of hydrolysis that occurred was determined to only be 3% of the PMES side groups, confirming that the majority of the repeat units remained intact.

**Figure 2:**
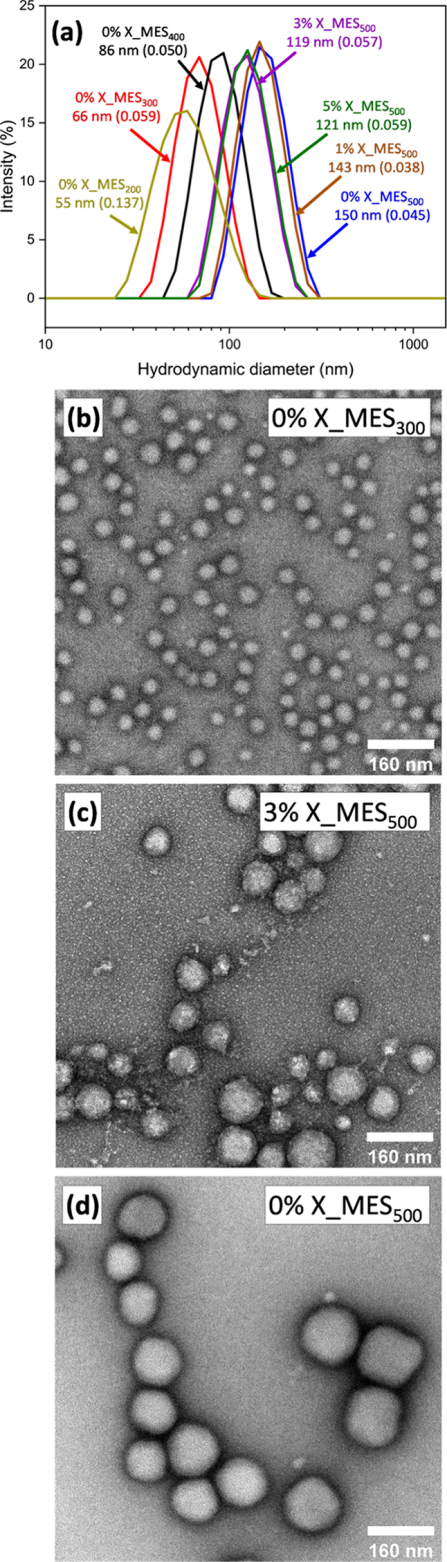
PKSPMA_50_–P(MES_*m*_-EGDMA_1–*m*_)_*y*_ diblock copolymer nanoparticles prepared via RAFT-mediated polymerization of MES in water at 70 °C (20% w/w, pH 2.0). (a) Corresponding DLS intensity-average size distributions at pH 2 (25 °C, 0.10% w/w, 1 mM KCl; the number in parentheses represents the DLS polydispersity index). Representative TEM images of nanoparticles: (b) 0% X_ MES_300_, (c) 3% X_ MES_500_, and (d) 0% X_ MES_500_.

A series of anionic PKSPMA_50_-PMES_*y*_ diblock copolymers were then prepared at pH 2 at 20% w/w, targeting core-forming block DPs ranging from 100 to 500. In all cases, more than 99% MES conversion was achieved within 10 h at 70 °C, as judged by ^1^H NMR spectroscopy. For PKSPMA_50_-PMES_100_ a translucent gel was obtained (Figure S5a), potentially suggesting the formation of worm-like micelles, as reported in related PISA formulations.^36^ However, upon analysis of this copolymer via TEM and DLS, no evidence of self-assembled nanoparticles was obtained. Therefore, the observed gel is simply a result of molecularly entangled chains. On increasing the target PMES DP to 200, a milky dispersion was obtained, indicating the formation of self-assembled nanoparticles. This was confirmed by DLS (Figure 2a). However, the size distribution obtained was relatively broad. For PMES DPs ≥ 300, milky white dispersions were obtained (Figure S5) and DLS particle size distributions for 0.1% w/w dispersions at pH 2 were monomodal and relatively narrow (Figure 2a). As expected for this PISA system, increasing the core-forming block DP caused larger nanoparticles to be obtained, with the mean hydrodynamic diameter increasing from 66 nm for PKSPMA_50_-PMES_300_ to 150 nm for PKSPMA_50_-PMES_500_. Furthermore, TEM analysis of the nanoparticles confirmed the formation of spherical micelles (Figure 2b,d), with no evidence seen of higher-order morphologies such as worm-like micelles or vesicles. This was expected due to this being an RAFT emulsion polymerization formulation and the highly anionic nature of the PKSPMA_50_ electrosteric stabilizer block. Specifically, the low solvation of the growing cores and high interparticle repulsion prevents sphere–sphere fusion and results in kinetically trapped spheres. Thus, in the present study, only spherical nanoparticles were observed. In addition, this observation further supports the hypothesis that the PKSPMA_50_-PMES_100_ sample did not reach the critical point for well-defined self-assembled structures to form.

Given that PMES contains carboxylic acid groups, it was expected that these nanoparticles would swell upon increasing the pH (Figure 1c). Thus, in order to investigate the effect of core cross-linking on nanoparticle formation and degree of swelling, an additional series of samples were prepared where EGDMA was added as a core cross-linking comonomer at 1, 3, and 5 mol % during MES polymerization when targeting an overall DP of 500 [referred to herein as *n*% X_MES_*y*_, where *n* represents the mol % of EGDMA used and *y* represents the target DP of PMES]. For these cross-linked particles, the DLS size distributions remained relatively narrow (Figure 2a) but the mean particle diameters decreased as the EGDMA content was increased. This is presumably because cross-linking lowers the mobility of the in-situ-generated block copolymer chains and therefore inhibits chain exchange, resulting in a lower copolymer aggregation number per particle (and hence smaller particle size).^35^ TEM analysis of the cross-linked particles confirmed that spherical nanoparticles were formed with the observed diameters, in agreement with DLS measurements (Figure 2c).

**Figure 3:**
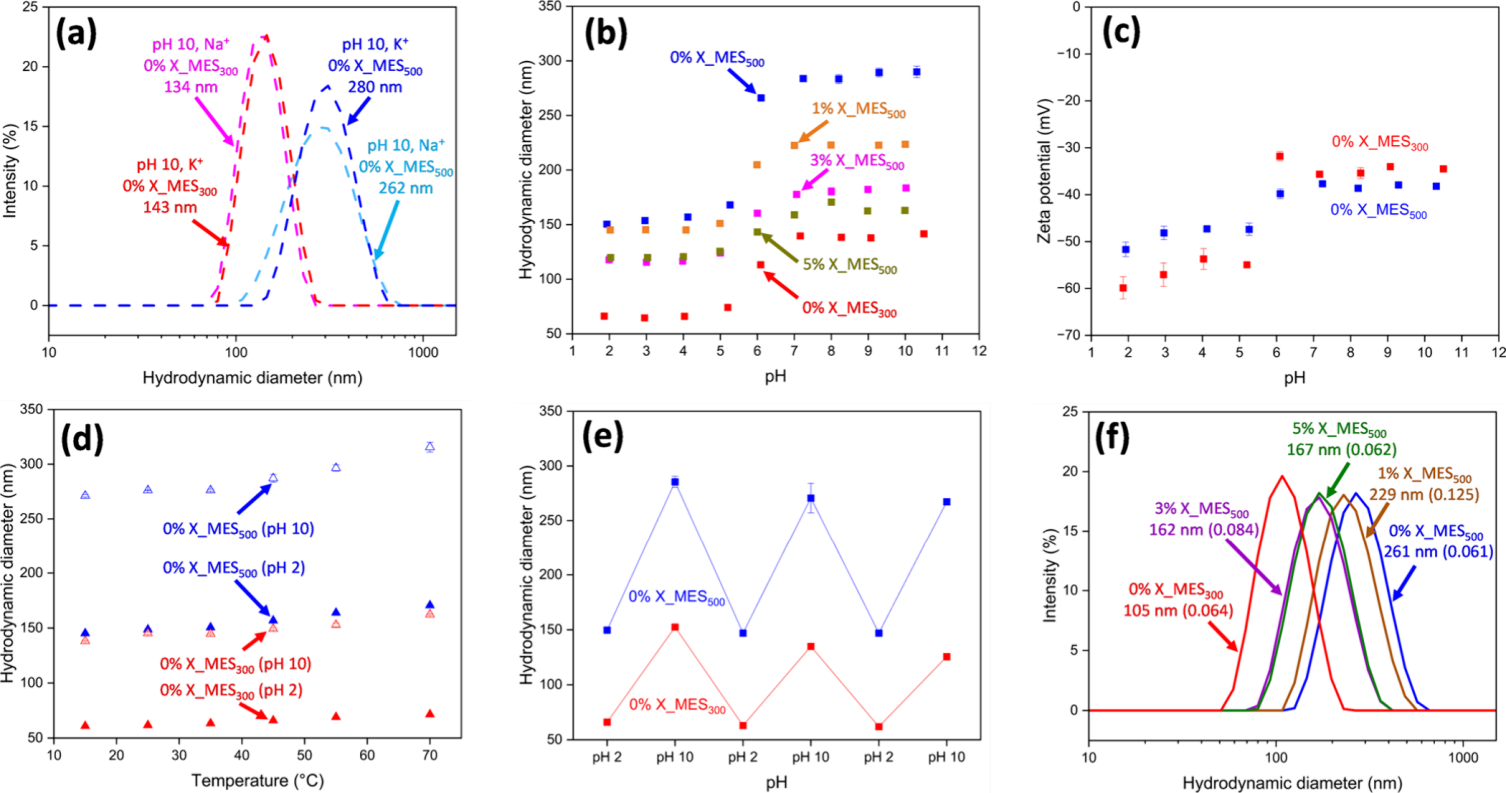
(a) DLS intensity-average size distributions for 0% X_MES_*y*_ dispersions (25 °C; 0.10% w/w, 1 mM NaCl or 1 mM KCl) at pH 10 (adjusted with 1 M NaOH or 1 M KOH). (b) Hydrodynamic diameter vs pH for *n*% X_MES_*y*_ dispersions (25 °C; 0.10% w/w, 1 mM KCl). (c) Zeta potential vs pH for 0% X_MES_*y*_ dispersions (25 °C; 0.10% w/w, 1 mM KCl). (d) Hydrodynamic diameter vs temperature for 0% X_MES_*y*_ dispersions (0.10% w/w, 1 mM KCl) at pH 2 or pH 10. (e) Variation in the particle size with pH cycling for 0% X_MES_*y*_ dispersions (25 °C; 0.10% w/w, 1 mM KCl). (f) DLS intensity-average size distributions for *n*% X_MES_*y*_ dispersions (0.10% w/w) diluted with 80/20% w/w methanol/water.

The 0% X_MES_*y*_ nanoparticles were found to swell upon increasing the pH of the aqueous phase to 10 without molecularly dissolving (Figure 3a). This is perhaps unexpected, as without the presence of cross-linker the doubly anionic copolymer chains should be soluble in water and thus cause nanoparticle disassembly. However, this was not the case and suggests that some in situ core-cross-linking takes place during RAFT emulsion polymerization, which has previously been observed for related formulations.^23^ Similarly, when challenged with methanol, the particles prepared in the absence of EGDMA were found to swell and not disassemble (Figure 3f). The swollen particle size distributions remained monomodal, and the swelling ratio of the 0% X_MES_*y*_ particles at pH 10 was approximately 2.0 (Table S1). Interestingly, while the solubility of monomer slightly differs in the presence of NaOH and KOH (Figure S3), the swollen particle diameters are the same regardless of the base used to change the pH (Figure 3a).

Subsequently, the 0% X_MES_*y*_ nanoparticles were titrated through the addition of KOH and the mean hydrodynamic diameter and zeta potential were monitored at 25 °C. The prepared MES-containing particles underwent sharp volume transitions upon increasing the pH above ∼5 (Figure 3b). For example, the average diameter of 0% X_MES_300_ was 66 nm below pH 5.0, underwent a sharp increase in size between pH 5.0 and 7.0, and reached a mean diameter of 143 nm at pH ≥ 7.0. Similarly, the swelling behavior of 0% X_MES_500_ followed the same trend but with larger values (150 nm < pH 5.0 and 280 nm ≥ pH 7.0). This is due to the carboxylic acid groups within the nanoparticle cores becoming deprotonated and thus causing swelling, confirming that these nanoparticles behave as nanogels.

The observed swelling transition correlates well with the previously reported p*K*_a_ value of 5.4 for PMES homopolymer.^31^ As expected, the zeta potential for all particles remained negative at all pH values due to the presence of the highly anionic PKSPMA_50_ stabilizer (Figure 3c). It is noteworthy that the zeta potential values measured herein are derived from the electrophoretic mobility and that the calculation of zeta potential is based on several assumptions.^37^ Unexpectedly, the measured zeta potential became less anionic on increasing the pH. The reason for this is not entirely apparent, as one would expect the zeta potential to become more anionic with the deprotonation of carboxylic acid groups within the nanoparticle cores. This observation could potentially be attributed to the increase in the volume of the nanoparticles upon swelling, affecting their electrophoretic mobility when compared to unswollen particles. Nevertheless, the zeta potential of the swollen nanogels remained less than −30 mV, and the nanogels remained highly monodisperse and nonaggregated over a wide pH range (Table S1).

The effect of temperature on the size of the non-cross-linked nanoparticles at both pH 2 and 10 was investigated by increasing the temperature of the diluted dispersion from 15 to 70 °C. According to Figure 3d, increasing the temperature very slightly increases the particle diameter at both pH values. However, the pH-responsiveness of the nanoparticles is not affected by temperature, with markedly increased sizes being observed at pH 10 regardless of temperature. This moderate change in size on heating is reversible on cooling back to 25 °C and occurs consistently over multiple heating–cooling cycles (Figure S6a). The relatively small swelling ratio on increasing the temperature (Table S2) is consistent with PMES not having a reported lower critical solution temperature (LCST) or upper critical solution temperature (UCST). In addition, highly negative zeta potential values of approximately −40 mV are maintained between 15 and 70 °C (Figure S6b), which further demonstrates the colloidal stability of these nanogels.

To investigate the reversibility of the volume change of the 0% X_MES_*y*_ nanogels on varying the pH, three successive shrink/swell cycles were conducted (Figures 3e and S7). The size change of the nanogels was highly reversible, with the measured particle diameter at low pH (unswollen) remaining consistent across the cycles and the nanogels becoming swollen on increasing the pH. As expected, the swollen diameter of nanogels decreases slightly on repeatedly cycling the pH due to the buildup of ionic strength in the dispersion.

The addition of EGDMA did not affect the pH at which the *n*% X_MES_500_ nanoparticles (where *n* = 1, 3, and 5) become swollen (Figure 3b). However, the addition of EGDMA caused the swelling ratio of the *n*% X_MES_500_ nanogels to decrease from 1.9 for 0% X_MES_500_ to 1.3 for 5% X_MES_500_ (Table S1). This decrease in swelling ratio demonstrates that core-cross-linking has been successful and serves as a useful way to attenuate the degree of swelling for these nanogels. Similarly, the *n*% X_MES_500_ nanogels remain intact and monomodal in size but become swollen when challenged with 80/20% w/w methanol/water (Figure 3f).

## Conclusions

In summary, colloidal nanogels with narrow particle size distributions were prepared via polymerization-induced self-assembly (PISA) using a previously unreported pH-responsive polysuccinate core and a poly(sulfonate)-functional corona. Specifically, PKSPMA_50_ was chain extended with MES using RAFT aqueous emulsion polymerization at pH 2 and 20% w/w. This PISA formulation allows the particle size to be readily controlled by a systematic variation of the target DP of the PMES core-forming block. Colloidally stable diblock copolymer nanoparticles were obtained when targeting relatively high core-forming block DP (≥300) and targeting shorter PMES blocks led to ill-defined self-assembled structures or chain entanglement. The highly anionic nature of these nanogels imparted by the PKSPMA stabilizer means that they remain colloidally stable over a wide pH range. Furthermore, well-defined pH-responsive nanogels were prepared even in the absence of a cross-linker, and the addition of a core cross-linking comonomer led to smaller nanogels with lower swelling ratios being formed. These nanogels reversibly change in size on varying the pH above and below pH 5–7 but do not show any significant response to temperature. On the whole, this work provides a demonstration of MES, a succinate-functional monomer, as a novel core component in the preparation of pH-responsive nanogels via PISA. Thus, offering a versatile platform for future nanogel formulations and applications thereof, for example in the preparation of polymer nanoparticle complex coacervate gels.^23^

## Supporting Information Available

Experimental materials and methods; ^1^H NMR spectra and GPC chromatograms of PKSPMA_50_ macro-CTA; solubility test of MES monomer; kinetic study of PKSPMA_50_-MES_300_ synthesis; digital photograph of PKSPMA_50_-MES_*y*_ reaction products; additional temperature- and pH-responsive behavior of PKSPMA_50_-MES_300_ and PKSPMA_50_-MES_500_ nanoparticles; and supporting data tables for nanoparticle diameter and volume swelling ratios (PDF)
