# Supplementary material for: pH-Responsive Nanogels Generated by Polymerization-Induced Self-Assembly of a Succinate-Functional Monomer
Source: Macromolecules. 2024 Apr 8;57(8):3496–501. doi: 10.1021/acs.macromol.4c00427 (PMC11044572; doi:10.1021/acs.macromol.4c00427)
Supplement: Supplementary file 1 — ma4c00427_si_001.pdf [file ma4c00427_si_001.pdf]

Supporting Information

pH-responsive nanogels generated by polymerization-induced self-assembly of a succinate-functional monomer

Ruiling Du,[a,b] Lee A. Fielding*[a,b]

[a] Department of Materials, School of Natural Sciences, University of Manchester, Oxford Road, Manchester, M13 9PL, UK.

[b] Henry Royce Institute, The University of Manchester, Oxford Road, Manchester, M13 9PL, UK.

* lee.fielding@manchester.ac.uk

Materials and Methods

Materials: 3-sulfopropyl methacrylate potassium salt (KSPMA, 98 %), mono-2-(methacryloyloxy)ethyl succinate (MES, 95 %, inhibited with 500 ppm MEHQ) and 4,4′-azobis(4-cyanovaleric acid) (ACVA, 98 %) were purchased from Sigma-Aldrich (UK) and used as received without further purification. Methanol and 1,4-dioxane purchased from Sigma-Aldrich (UK) were organic synthesis grade. Ethylene glycol dimethacrylate (EGDMA, 98%, inhibited with 100 ppm MEHQ) was purchased from Alfa Aesar (UK). 4-Cyano-4-(2-phenylethane sulfanylthiocarbonyl) sulfanylpentanoic acid (PETTC) was synthesized in-house using previously reported protocols [1]. Deuterated NMR solvents (D_2O and methanol-d_4) for NMR characterization were purchased from Cambridge Isotope Laboratories (UK). KOH, NaOH and HCl (~ 37%) were purchased from Fisher Scientific (UK) and then diluted in-house to adjust solution pH. Dialysis tubing (regenerated cellulose, MWCO = 3.5 kDa, diameter = 29 mm) was received from Fisher Scientific (UK). Deionized water was obtained from an in-house SUEZ Purite Analyst water purification unit.

Synthesis of PKSPMA$_{50}$ *via* RAFT solution polymerization: The preparation of PKSPMA by RAFT solution polymerization has been reported before.[1] The protocol used for the preparation of PKSPMA$_{50}$ is as follows. KSPMA monomer (19.0 g, 77.1 mmol), PETTC RAFT agent (523.7 mg, 1.5 mmol, dissolved in dioxane), ACVA (86.5 mg, 0.3 mmol, PETTC/ACVA molar ratio = 5), and pH 5.5 acetate buffer (82.9 g, final buffer/dioxane ratio = 3) were weighed into a 250 ml round-bottomed flask, which was then sealed and purged with N_2 for 30 min. The sealed flask was placed in a preheated water bath at 70 °C for 90 min. The reaction was quenched by immersion in an ice bath and opening the flask to air. The resulting PKSPMA was purified by dialysis against 10:1 water/methanol overnight. The purified solution was dried under vacuum at 30 °C to evaporate volatiles and then freeze-dried from aqueous solution overnight to yield a yellow product (61.2 % yield). The mean degree of polymerization (DP) of PKSPMA was calculated to be 50, as determined by ^{1}H-NMR using D_2O.

Preparation of PKSPMA$_{50}$-P(MES$_m$-co-EGDMA$_{1-m}$)$_y$ block copolymer nanoparticles *via* RAFT aqueous emulsion polymerization: A typical polymerization using a (MES & EGDMA) : PKSPMA$_{50}$: ACVA ratio of 500 : 1: 0.25 at 20 % w/w solids with a MES : EGDMA molar ratio of 97 : 3 at pH 2 water is as follows.

$PKSPMA_{50}$ (0.2 g, 0.019 mmol), MES (2.087 g, 9.065 mmol), EGDMA (55.6 mg, 0.280 mmol), and ACVA (1.3 mg, 4.67 μmol; CTA/initiator molar ratio = 4.0) were weighed into a 25 mL round bottomed flask. Deionized water and 0.25 M HCl were added to adjust the mixture to pH 2 to prevent ionization of the -COOH groups on MES during the polymerization and to produce a 20% w/w aqueous mixture. The solution was then purged with N_2 for 30 min prior to immersion in a water bath set at 70 °C. The heated reaction was stirred for 10 h before the polymerization was quenched by cooling in an ice bath and exposure to air. Monomer conversions were determined *via* ^{1}H NMR using a methanol-d_4/D_2O mixture (80/20 % w/w).

Proton nuclear magnetic resonance (^{1}H NMR) spectra were recorded on a Bruker Advance III 400 MHz spectrometer with 128 scans averaged per spectrum at 25 °C.

Aqueous gel permeation chromatography (GPC) measurements were conducted using a phosphate buffer eluent (pH 9) containing 30 % v/v methanol at a flow rate of 1.0 mL min^{-1} at ambient temperature. The instrument was equipped with two PL aquagel-OH MIXED-H 8 μm columns and a refractive index detector (Shodex RI-101) was used to assess molar mass distributions. The system was calibrated with a series of near-monodisperse poly(ethylene oxide) standards. Samples were prepared in the phosphate buffer eluent at 2 mg mL^{-1}.

Dynamic light scattering (DLS) studies were conducted using a Malvern Zetasizer Ultra instrument to measure both hydrodynamic diameter (D_h) and zeta potential. The instrument is equipped with a He–Ne solid-state laser operating at 633 nm, detecting back-scattered light at a scattering angle of 173°. All samples were diluted to 0.1 % w/w using 1.0 mM KCl or 1.0 mM NaCl as background electrolyte and data were averaged over three consecutive runs. Disposable folded capillary cells (Malvern DTS1070) were used for measuring both D_h and zeta potential. The dispersion pH was adjusted manually from pH 2 to pH 10 using KOH or NaOH (0.25 M/0.025 M) and HCl (0.25 M/0.025 M). The dispersion temperature was automatically adjusted from 15 °C to 70 °C by the Malvern Zetasizer Ultra instrument. All DLS samples were equilibrated for 240 seconds before measurements.

Transmission electron microscope (TEM) observations were carried out on a FEI Tecnai G2 F20 instrument operating at an accelerating voltage of 200 kV and connected to a Gatan 1k CCD camera. Copper/palladium TEM grids (Agar Scientific, UK) were surface coated with a thin film of amorphous carbon, then subjected to a plasma glow discharge for 30 seconds to produce a hydrophilic surface. The obtained dispersions were diluted from 20% w/w to 0.1% w/w solids at pH 2. A hydrophilic grid was placed onto an aqueous droplet (40 μL) of a 0.1 w/w dispersion for 1 min and then blotted with filter paper to remove excess solution. This grid was then negatively stained *via* placing onto a uranyl acetate solution (0.5 wt %) droplet (40 μL) for 30 seconds. Excess stain was removed by blotting and the grid was carefully dried with a vacuum hose.

Supporting Figures

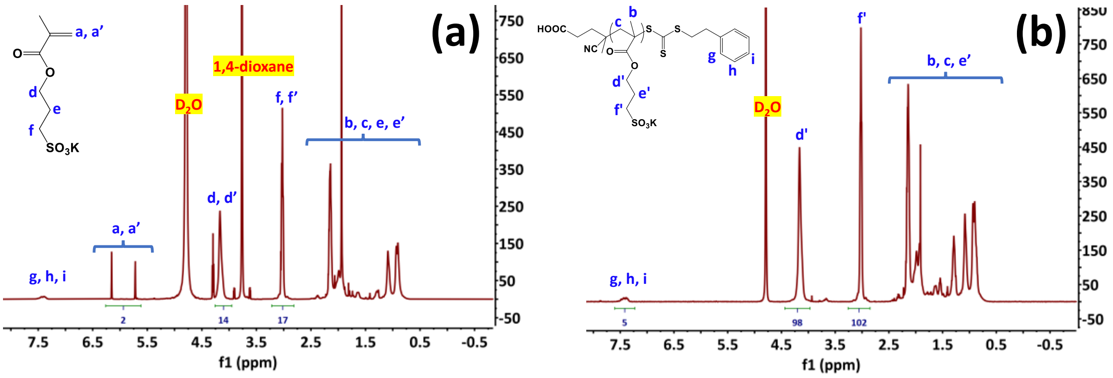

Figure S1. Assigned ^{1}H NMR spectra of (a) an unpurified PKSPMA reaction product and (b) a purified and freeze-dried $PKSPMA_{50}$ macro-CTA. Both samples were dissolved in D_2O prior to analysis. The conversion for this reaction was calculated using unpurified PKSPMA reaction product spectra by comparing the integrated proton signals corresponding to the methacrylic polymer backbone at 2.83-3.26 ppm with that corresponding to the vinyl protons of the KSPMA monomer at 5.6 ppm-6.25 ppm. The degree of polymerization (DP) for the purified polymer was calculated by comparing the integrated proton signals corresponding to the methacrylic polymer backbone at 2.83- 3.26 ppm and 3.97-4.44 ppm with that corresponding to the aromatic protons of the chain end at 7.2-7.4 ppm.

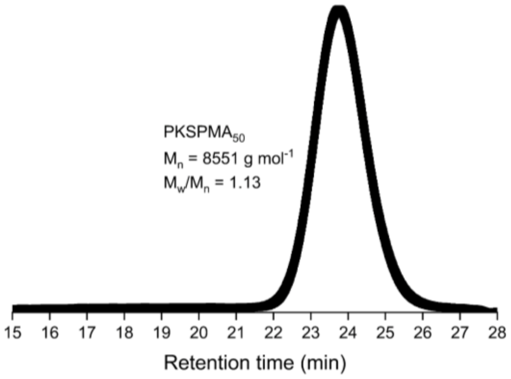

Figure S2. Aqueous gel permeation chromatography chromatogram obtained for $PKSPMA_{50}$ macro-CTA. A relatively narrow molecular weight distribution was achieved, suggesting successful RAFT polymerization.

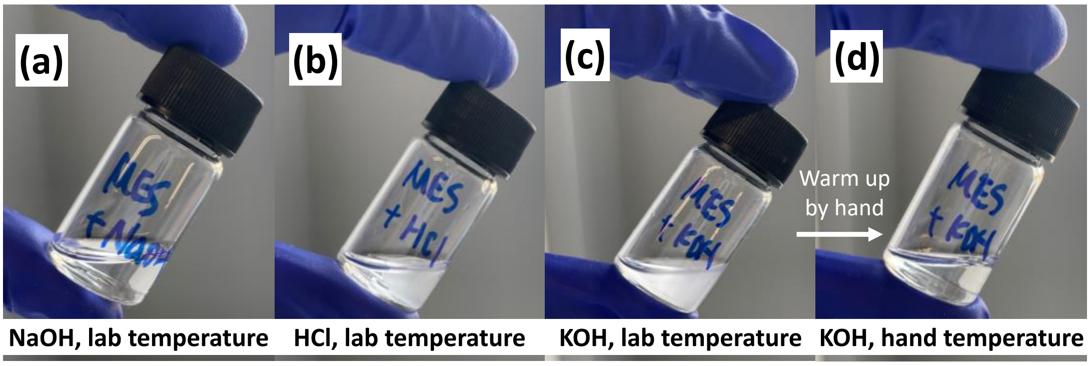

Figure S3. Photographs showing monomer solubility testing conducted by mixing 1 ml MES monomer with 1 ml (a) NaOH (1 M), (b) HCl (1 M), (c) KOH (1 M) at lab temperature and (d) KOH (1 M) after being warmed gently in a closed hand for approximately 5 minutes.

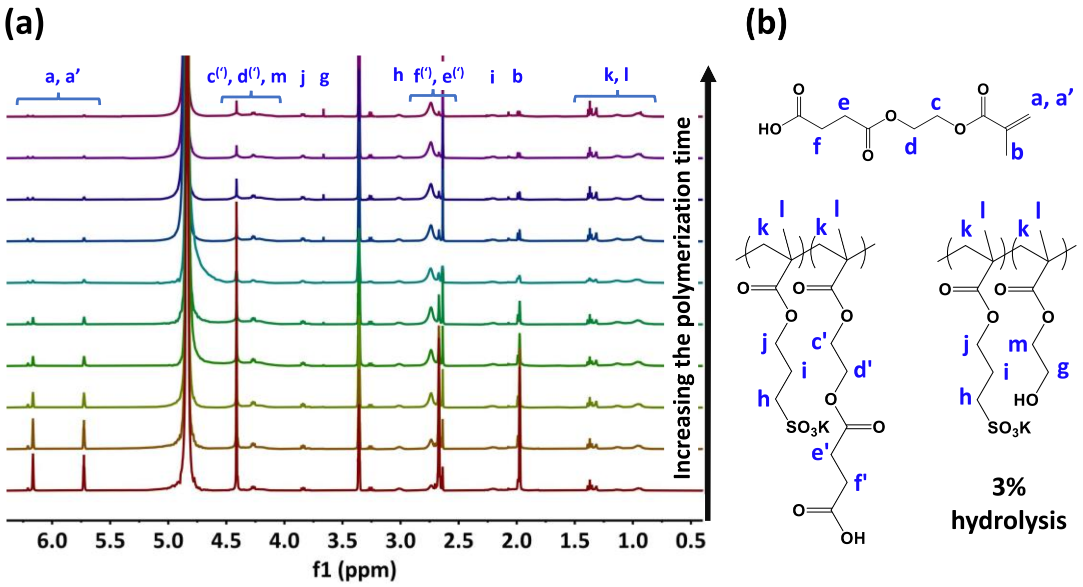

Figure S4. Kinetic studies for the RAFT emulsion polymerization of MES (target DP 300) using $PKSPMA_{50}$ as a macro-CTA in pH 2 water at 70 °C. (a) Assigned ^{1}H NMR spectra at different reaction time (samples were dissolved in 80/20 % w/w methanol-d_4/D_2O for measurements). (b) Chemical structures of MES monomer and the resulting diblock copolymer products.

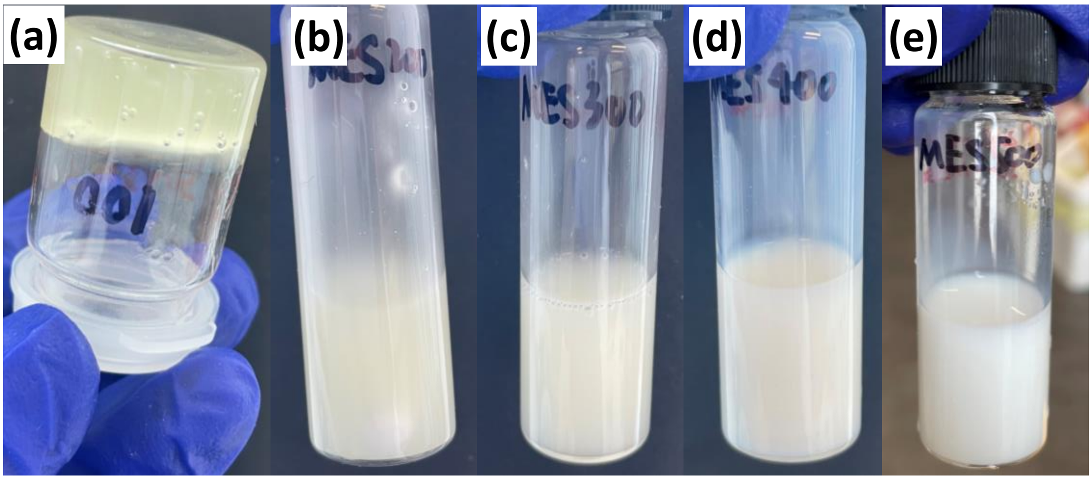

Figure S5. Photographs of the reaction products at room temperature after performing RAFT aqueous emulsion polymerizations of MES using $PKSPMA_{50}$ as a macro-CTA at pH 2, 70 °C, and 20% w/w. The target degree of polymerization of PMES was: (a) 100; (b) 200; (c) 300; (d) 400; (e) 500.

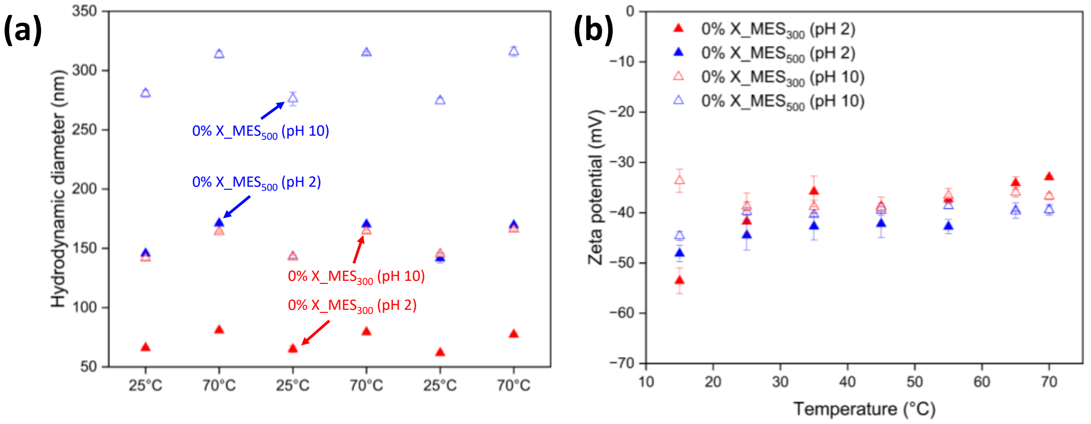

Figure S6. (a) Hydrodynamic diameter on cycling between 25 °C and 70 °C and (b) zeta potential vs. temperature for $PKSPMA_{50}$-$PMES_{300}$ (red triangles) and $PKSPMA_{50}$-$PMES_{500}$ (blue triangles) nanoparticles. All measurements were performed on 0.1% w/w copolymer dispersions prepared in the presence of 1.0 mM KCl background salt and the pH was adjusted using HCl or KOH.

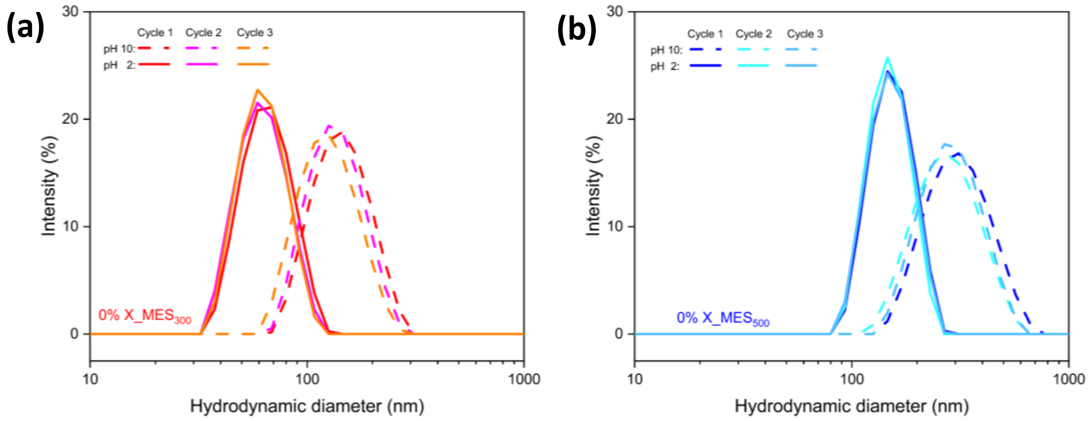

Figure S7. Particle size distributions for (a) $PKSPMA_{50}$-$PMES_{300}$ and (b) $PKSPMA_{50}$-$PMES_{500}$ nanoparticles on cycling the pH between 10 (dashed lines) and 2 (solid lines) for three cycles. All measurements were conducted at 25 °C for 0.1% w/w copolymer dispersions prepared in the presence of 1.0 mM KCl background salt.

Supporting Tables

Table S1. Particle diameter and volume swelling ratio for $PKSPMA_{50}$-$P(MES_{m}$-$EGDMA_{1-m})_{y}$ nanoparticles under various conditions. Particle diameters were measured by DLS and polydispersity index values are indicated in brackets.

Nanoparticle	$D_{h,pH2}$ / nm[a]	$D_{h,pH10}$ / nm[b]	$D_{h,MeOH}$ / nm[c]	Volume swelling ratio ($D_{h,pH10}$/ $D_{h,pH2}$)	Volume swelling ratio ($D_{h,MeOH}$/ $D_{h,pH2}$)
0% X_MES_{300}	66 (0.059)	142 (0.036)	105 (0.064)	2.2	1.6
0% X_MES_{500}	150 (0.045)	286 (0.077)	261 (0.061)	1.9	1.7
1% X_MES_{500}	143 (0.038)	224 (0.085)	229 (0.125)	1.6	1.6
3% X_MES_{500}	119 (0.057)	181 (0.012)	162 (0.084)	1.5	1.4
5% X_MES_{500}	121 (0.059)	160 (0.054)	167 (0.062)	1.3	1.4

[a] Measured using 1mM KCl as the dispersant and the pH was adjusted using HCl.

[b] Measured using 1mM KCl as the dispersant and the pH was adjusted using KOH.

[c] Measured using 80/20 % w/w methanol/water mixture as the dispersant. Instrument parameters (viscosity, refractive index, and dielectric constant values for methanol/water) used during measurements were as previous reported.[1]

Table S2. Particle diameter and volume swelling ratio of non-crosslinked nanoparticles at 15 °C and 70 °C. Particle diameters were measured by DLS and polydispersity index values are indicated in brackets.

Nanoparticle	$D_{h,15°C}$ / nm	$D_{h,70°C}$ / nm	Volume swelling ratio ($D_{h,70°C}$/ $D_{h,15°C}$)
0% X_MES_{300} (pH 2)	61 (0.035)	79 (0.051)	1.3
0% X_MES_{300} (pH 10)	138 (0.060)	165 (0.020)	1.2
0% X_MES_{500} (pH 2)	145 (0.067)	170 (0.018)	1.2
0% X_MES_{500} (pH 10)	271 (0.076)	315 (0.082)	1.2

References

(1) Wen, S.-P.; Saunders, J. G.; Fielding, L. A. Investigating the influence of solvent quality on RAFT-mediated PISA of sulfonate-functional diblock copolymer nanoparticles. *Polymer Chemistry* **2020**, *11* (20), 3416-3426.
